# The energy requirements and metabolic benefits of wilderness hunting in Alaska

**DOI:** 10.14814/phy2.13925

**Published:** 2018-11-14

**Authors:** Robert H. Coker, Melynda S. Coker, Larry Bartlett, Carl J. Murphy, Karolina Priebe, Timothy C. Shriver, Dale A. Schoeller, Brent C. Ruby

**Affiliations:** ^1^ Institute of Arctic Biology University of Alaska Fairbanks Fairbanks Alaska; ^2^ School of Management University of Alaska Fairbanks Fairbanks Alaska; ^3^ Pristine Ventures, Inc Fairbanks Alaska; ^4^ Isotope Ratio Core Laboratory University of Wisconsin‐Madison Madison Wisconsin; ^5^ Montana Center for Work Physiology and Exercise Metabolism University of Montana Missoula Montana

**Keywords:** Body composition, energy expenditure, hunter‐gatherer, intrahepatic lipid

## Abstract

The purported healthy aspects of subsistence foods have led to the popularity of the Paleo diet. There has been very little focus, surprisingly, on health benefits derived from the nomadic nature of humans during the Paleolithic era. The purpose of our study was to examine total energy expenditure (TEE), total energy intake (TEI), body composition, blood lipids, and intrahepatic lipid in humans during a 12‐day Alaskan backcountry expeditionary hunting (ABEH) immersion. Four healthy men (age: 42 ± 3 year, BMI: 27 ± 1 kg/m^2^) were recruited for the study. TEE was measured using the doubly labeled water method and a food diary was utilized to assess TEI. Body composition was measured using dual energy X‐ray absorptiometry (DXA); cross‐sectional area of the thigh (XT) and intrahepatic lipid (IHL) were measured using molecular imaging. Blood samples were collected for the measurement of blood lipids. DXA, XT, IHL, and blood data were collected pre‐ and immediately post‐ABEH. Results were analyzed using paired *t*‐tests and considered significant at *P* < 0.05. TEE and TEI averaged 18.1 ± 1.2 and 9.1 ± 2.5 MJ/day, respectively, indicating substantial negative energy balance (‐9.0 ± 1.3 MJ/day). There was a reduction in percent body fat (∆−3.3 ± 0.2%), total fat mass (∆−3.3 ± 0.4 kg), and visceral fat volume (Δ−261 ± 188 cm^3^). Lean tissue mass and XT was unchanged. There was a decrease in IHL (Δ−0.5 ± 0.1% water peak), and a trend (*P* = 0.055) toward reduction in LDL‐cholesterol. We conclude that constancy of physical activity during negative energy balance may provide metabolic benefits above and beyond variations in diet that exist with the hunter‐gatherer lifestyle.

## Introduction

*Homo erectus* and *Homo sapiens* remained entirely dependent on a hunter‐gatherer lifestyle for almost 2 million years (Hazarika 2007). Numerous epidemiological studies promote the important role of subsistence foods in the maintenance of metabolic health (Wahlqvist 2005). Unique archeological findings recovered at the Upward Sun River site represent the oldest human remains discovered on the American Side of Beringia or Alaska, showing strong linkage to the Paleo‐Arctic tradition (Potter et al. 2017).

Nutrient quality remains important in modern civilization, but the impact of the industrial revolution as it relates to physical activity and metabolic health has not been fully evaluated (Huneault et al. 2011; Forrester 2013). As a result, it is not entirely clear whether the obesity epidemic should be characterized as a disease or identified as an adaptive response to nutrient availability relative to overwhelming levels of physical inactivity (Chaput et al. 2012). Nutritional interventions that limit portion sizes and the intake of processed foods suggest the efficacy of dietary modification on improvements in metabolic health. However, these approaches may only dampen the adaptive response.

There are challenges in studying the independent influence of physical activity in hunter‐gatherer communities on metabolic health (Crittenden and Schnorr 2017). Additionally, the definition of exercise in Westernized society could be largely inconsistent with the activity required for subsistence without modernized vehicular transport. While useful in the well‐controlled laboratory setting (McArdle et al. 2001), prediction equations and/or exercise testing under variable load conditions with alterations in incline/decline fail to capture the true influence of constant physical/mental stress on energy expenditure. This has made it difficult to estimate caloric cost, and derive a better understanding of the health benefits enjoyed by the hunter‐gatherer lifestyle.

The use of doubly labeled water (DLW) has been utilized to quantify the total energy expenditure (TEE) during a wide variety of “field conditions” (Stroud et al. 1993; Schoeller 1999; Ruby et al. 2002; Tharion et al. 2004; Fallowfield et al. 2014; Margolis et al. 2014) and has been validated as the most accurate in free‐living humans (Potter et al. 2017). These data have helped provide a more valuable presentation of the metabolic and physical capabilities of humans. We hypothesized that a sustained mismatch between TEE and total energy intake (TEI) would occur in parallel with significant reductions in excess body fat, liver lipid, and blood lipids during a 12‐day Alaska backcountry expeditionary hunting trip (ABEH) for moose (*Alces alces*) and caribou (*Rangifer tarandus*).

## Materials and Methods

### Participants

Four middle‐aged males (Mean ± SB; 41.8 ± 2.5 years of age; body mass index [BMI] = 27.2 ± 1.1 kg/m^2^) were enrolled in this study. None of the participants were smokers and/or had been diagnosed with nor were symptomatic for cardiovascular, respiratory, neurological, or metabolic diseases. They were not taking any related medications. Immediately after obtaining informed consent, each participant completed a health history questionnaire. The pre‐ and post‐ABEH visits included the following assessments: (1) measurement of body weight, body composition, and molecular imaging of muscle and liver, (2) blood sampling, (3) measurement of exercise‐induced VO_2max_, and (4) measurement of TEE. TEI was estimated based on written dietary records for each meal or snack (Capling et al. 2017). The study and all related documents were reviewed and approved by the University of Alaska Fairbanks (UAF) Institutional Review Board.

### Hunting immersion

Four participants were recruited for the ABEH as arranged by Pristine Ventures, Inc and Shadow Aviation (Fairbanks, AK). In accordance with Alaska law, no individuals were guided, but hunters received basic instructions related to floating, paddling and load carriage, hiking and hunt preparation, and meat preservation. Bush plane transportation was provided to the hunting area. All participants completed the above noted measurements immediately prior to air departure to the field, and within 4 h of returning to Fairbanks, AK. Measurements occurred in the Clinical Research and Imaging Facility at University of Alaska Fairbanks (UAF). Inclement weather and complications with bush flight logistics precluded the collection and analysis of blood samples during LabCorp hours in participants #3 and #4. Due to potential shifts in background H_2_0 enrichment in hunters from the Mountain West (MW) compared to hunters from Alaska (AK), participants #2 (AK), and #3 (MW) received blank or dummy doses of DLW to correct for shifts during the ABEH as detailed in the DLW Administration and Urine Collection section below.

### Maximal oxygen uptake

Maximal O_2_ uptake (VO_2 max_) was determined using electronically instrumented bicycle ergometer (Lode, Amsterdam, Netherlands), a Parvomedics TrueOne 2400 integrated metabolic measurement system, and identical two‐minute work intervals of increasing intensity during the pre‐ and post‐ABEH sessions (Walter et al. 2005).

### Body composition and intrahepatic lipid

All body composition and imaging measurements were taken with participants wearing the same lightweight clothing (“scrubs”), and all jewelry was removed. All four participants completed pre‐ and post‐ABEH measurements of body composition via a dual‐energy X‐ray absorptiometry (GE iDXA) scan. During the DXA scan, participants were instructed to lie motionless in the supine position on the iDXA table. The hands and arms of the participants were placed parallel to but not touching the body. In order to diminish foot movement and accurate placement of feet and legs, a Velcro strap was secured around both ankles. The iDXA was calibrated at least 3 × times/week using the epoxy resin phantom.

Measurements of the cross‐sectional area of the upper thigh muscles (XT) and intrahepatic (IHL) was obtained using a Toshiba Excelart/Vantage 1.5 T magnetic resonance/spectroscopy imaging system (Canon, Õtawara, Tochigi, Japan). Axial and coronal T1‐weighted images were acquired using a Field Echo sequence (TR = 172 msec, TE = 4 msec), and Axial T2 images were acquired with a Fast Spin Echo sequence (TR = 3700 msec, TE = 90 msec). All images were acquired on a QD knee coil after having rested in the supine position for at least 30 min (Berg et al. 1993). The same technician maintained the same body position and obtained the scans in the same axial plane during all measurements. During each visit, seven of the axial T1‐weighted images (FOV = 30 × 30 cm, matrix 256 × 256, NAQ = 2) were selected based on the half‐way point of the femur or between the superior border of the patella and the greater trochanter. These images were analyzed using Osirix software (Pixmeo, Bernex, Switzerland) (Hulmi et al. 2009).

Participants were placed in a prone, head‐first position in the whole body coil of the MRI system for the measurement of IHL (Bennett et al. 2012). Axial and coronal T1‐weighted images were acquired using a Field Echo sequence (TR = 172 msec, TE = 4 msec), and Axial T2 images were acquired with a Fast Spin Echo sequence (TR = 3700 msec, TE = 90 msec, FOV = 30 × 50 cm). On return visits, initial images were referenced to ensure consistent voxel placement. Spectra were acquired on a 3 × 3 × 2 cm voxel using a PRESS sequence (TR = 2000 msec, TE = 136, NAQ = 256).

Raw data from the spectra, including an un‐suppressed water reference, were converted to ascii format using a custom script before analysis using the jMRUI software. All spectra were Fourier transformed, phased, and referenced (1.4 ppm for lipid spectra, 4.8 ppm for water reference). The signals were fit using the AMARES non‐linear‐least‐squares algorithm within jMRUI. The results from both the lipids spectra and water reference spectra were then used to calculate a lipid‐to‐water ratio (Bennett et al. 2012).

### DLW administration and urine collection

**Figure 1:**
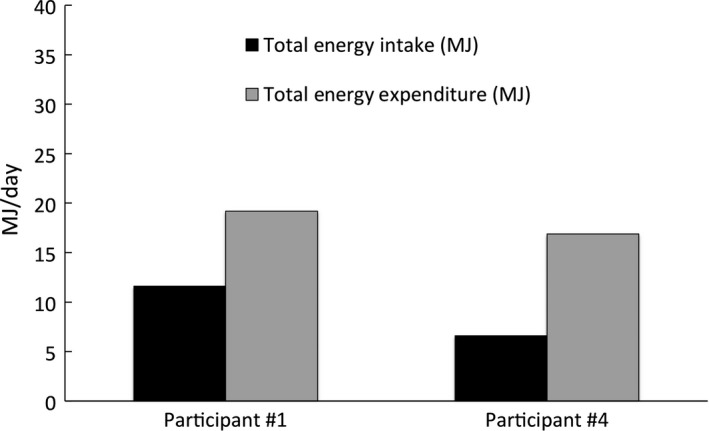
Total energy expenditure and total energy intake in participant #1 from Alaska and participant #4 from the Mountain West. Data are presented as mean values for participant #1 and #4.

Two of the participants were Alaska residents and two participants originated from the contiguous United States. The two participants from the MW were exposed to an Alaska hunt site water source that differed dramatically from that of their usual domicile (Schoeller et al. 1986). Therefore, one participant from each location (i.e., #2 and #3 originating from AK and the MW, respectively) were randomly assigned to either a true dose or a “sham dose” of DLW to adjust for potential alterations in background enrichments of ^18^O and ^2^H. All “true dose” participants provided a baseline urine sample for determination of background H_2_O enrichment, and then received an oral dose of doubly labeled water (0.39 g·kg estimated TBW^−1^ H_2_^18^O, 0.23 g·kg estimated TBW^−1^ ^2^H_2_O, Cambridge Isotope Laboratories, Andover, MA, Isotec, Inc., Miamisburg, OH) or a sham dose. The initial dose was given upon arrival to Fairbanks in the evening hours at ~22:00 h as described previously (Ruby et al. 2002). These doses are consistent with other scenarios involving high levels of energy expenditure (Schoeller et al. 2000; Pontzer et al. 2015). To ensure complete isotopic delivery, dose vials were rinsed three times with ~30 mL of water and consumed by participants. Subsequent urine samples were collected throughout the ABEH according to the schedule presented in Figure 1.

All urine samples were collected in sterile, polypropylene, nonpyrogenic, RNase/DNase‐free tubes (Corning, Inc., Corning NY) and wrapped in Parafilm™ (Bernis NA, Nina, WI). A precise description of dosing and sampling intervals has been provided in Table 1. Temperature conditions ranged from 4°C to 10°C, and samples were kept in dry storage during the event.

**Table 1:** Isotope dosing and sample collection protocol

Participant #1									
Day	−1	0	2	3	7	10	13	13	14
Collection times	17:45	05:40	06:25	07:15	06:00	06:30	06:15	21:30	06:30
Dose information									
0.39 g H_2_^18^O, 0.23 g ^2^H_2_O/kg TBW 2.0 g ^2^H_2_O	*								
Urine sampling									
Background	*								
First void		*	*	*	*	*	*		
Second void Urine sample collection for Participant #2 was similar to Participant #1 but included dummy dose of DLW		*							*

Participant #4									
Day	−1	0	2	3	7	10	13	13	14
Collection times	17:45	05:30	06:30	06:30	06:30	06:30	06:30	21:00	05:00
Dose information									
0.39 g H_2_^18^O, 0.23 g ^2^H_2_O/kg TBW 2.0 g ^2^H_2_O	*								
Urine sampling									
Background	*								
First void		*	*	*	*	*	*		
Second void Urine sample collection for participants #3 was similar to Participant #4 but included dummy dose of DLW		*							*

Analysis of isotopic enrichment and calculation of TEE. All analyses were conducted at the Isotope Ratio Core Laboratory at the University of Wisconsin, Madison (Hoyt et al. 1991). To obviate any issues for the baseline abundances, we employed information from the placebo subjects on the absolute value of the abundances by fitting to an exponential model [*δ*_i_ = (*δ*_o_−*δ*_inf_)^−kt^], where *δ*_i_ is the permil value measured at time i, *δ*_o_ is the permil value of the baseline urine collected before relocating to the hunt site, *δ*_inf_ is the permil value at infinite time after arriving at the hunt site, *k* is the exponential rate constant initially estimated from the two participants receiving DLW, and *t* is the time after arriving at the hunt site. The *δ*_inf_ values were −11.6 (−11.0, −12.2) and −117.6 (−116.8, −118.0), respectively, for ^18^O and ^2^H versus SMOW‐V2 (Standard Mean Ocean Water‐Vienna 2) (Ruby et al. 2002). Following the theoretical construct described by Stroud et al. (1993), we used the enrichments calculated relative to the baseline abundance at infinite time to calculate elimination rate (Speakman 1995). Total body water was calculated using the measured immediate, pre‐dose baseline specimen and abundance after overnight equilibration. CO_2_ production was calculated as summarized by Thorsen et al. (2011), assuming a respiratory exchange ratio of 0.86 to calculate TEE. Isotopic abundances were measured by isotope ratio mass spectrometry utilizing equilibration with CO_2_ for oxygen and chromium reduction for hydrogen (Ruby et al. 2002).

Prior to analysis of deuterium abundance, dry carbon was utilized to clean urine samples and clean fluid was reduced over chromium at 850°C. A Finnigan MAT Delta Plus isotope ratio mass spectrometer (Thermo Finnigan, San Jose, CA) was used to conduct the analysis of deuterium abundance (Hoyt et al. 1991). In order to measure the ^18^O/^16^O ratio, the second urine sample was equilibrated with 1 mL of STP of CO_2_ at 25°C (Fujita et al. 2007). Water turnover was calculated from the ^2^H dilution space and elimination space where rH_2_O = N_d_ + *k*_d_. N_d_ is the dilution space measured using ^2^H_2_ and *k*_d_ is the fractional turnover rate of ^2^H after equilibration (Fjeld et al. 1988). All analyses were performed in duplicate, and all urine specimens from each participant were analyzed during the same batch.

### Statistical analysis

Data were analyzed using Microsoft Excel, iDXA, Osirix, and Prism 5 software. Data are presented as means ± SD. Paired *t*‐tests were utilized to compare differences in pre‐ABEH and post‐ABEH data. Data were compiled from all four participants with two exceptions. Data related to energy expenditure/energy intake include only participants #1 and #4 due to the influence of changes in water sources on background enrichments. Logistical conflicts with outbound bush flights precluded blood sampling in participants #3 and #4. As a result, blood sampling and analysis was performed only in participants #1 and #2. Statistics were considered significant with a *P*‐value of less than 0.05.

## Results

Weather conditions ranged from 4°C to 16°C and all four participants paddled and traversed ~200 river kilometers over the course of the entire trip. Animal harvests were taken on days 5 and 7 for participants #1 and #2, totaling ~500 kg. Day 4 harvests for participants #3 and #4 totaled ~100 kg.

Total energy expenditure and total energy intake. These data are provided in Figure 1. TEE was 19.2 MJ/day in participant #1 and 16.9 MJ/day in participant #4. TEI was 11.6 MJ/day in participant #1 and 6.6 MJ/day in participant #4. Caloric deficit was 7.7 MJ/day and 10.3 MJ/day in participants #1 and #4, respectively. Overall, TEE, TEI, and caloric deficit were 18.1 MJ/day, 9.1 MJ/day, and 9.0 MJ/day, respectively. The average rH_2_O values were 4.7 L/day^−1^ and 2.5 L/day^−1^ for participants #1 and #4, respectively.

### Aerobic capacity

There were no significant changes in relative VO_2max_ from pre‐ (36.3 ± 5.4 mL/kg^−1^/min^−1^) to post‐ABEH (39.4 ± 5.3 mL/kg^−1^/min^−1^) or absolute VO_2max_ from pre‐ (3.4 ± 0.4 mL/kg^−1^/min^−1^) to post‐ABEH (3.5 ± 0.4 mL/kg^−1^/min^−1^) (Table 2).

**Table 2:** Anthropometrics and fitness

	Pre	Post	∆
Weight			
#1	89.5	87.0	−2.5
#2	85.0	83.0	−2.0
#3	105.9	101.8	−4.1
#4	93.3	89.5	−3.8
Mean ± SD	93.5 ± 7.8	90.3 ± 7.0	−3.1 ± 0.9^a^
Body mass index (kg/m^2^)			
#1	26.4	26.4	0.0
#2	27.3	26.6	−0.6
#3	29.0	27.9	−1.1
#4	26.3	25.2	−1.1
Mean ± SD	27.3 ± 1.1	26.6 ± 1.0	−0.7 ± 0.4^a^
Maximal oxygen consumption (mL/kg^−1^/min^−1^)			
#1	42.7	43.5	0.8
#2	38.6	40.1	1.5
#3	27.8	30.5	2.7
#4	36.0	43.5	7.5
Mean ± SD	36.3 ± 5.4	39.4 ± 5.3	3.1 ± 2.6
Maximal oxygen consumption (L/min^−1^)			
#1	3.82	3.78	−0.04
#2	3.28	3.33	0.05
#3	2.94	3.10	0.16
#4	3.36	3.89	0.53
Mean ± SD	3.4 ± 0.3	3.5 ± 0.3	0.2 ± 0.2

Data are presented as means ± SD.

a Represents significant difference (*P* < 0.05).

### Body weight and composition

**Figure 2:**
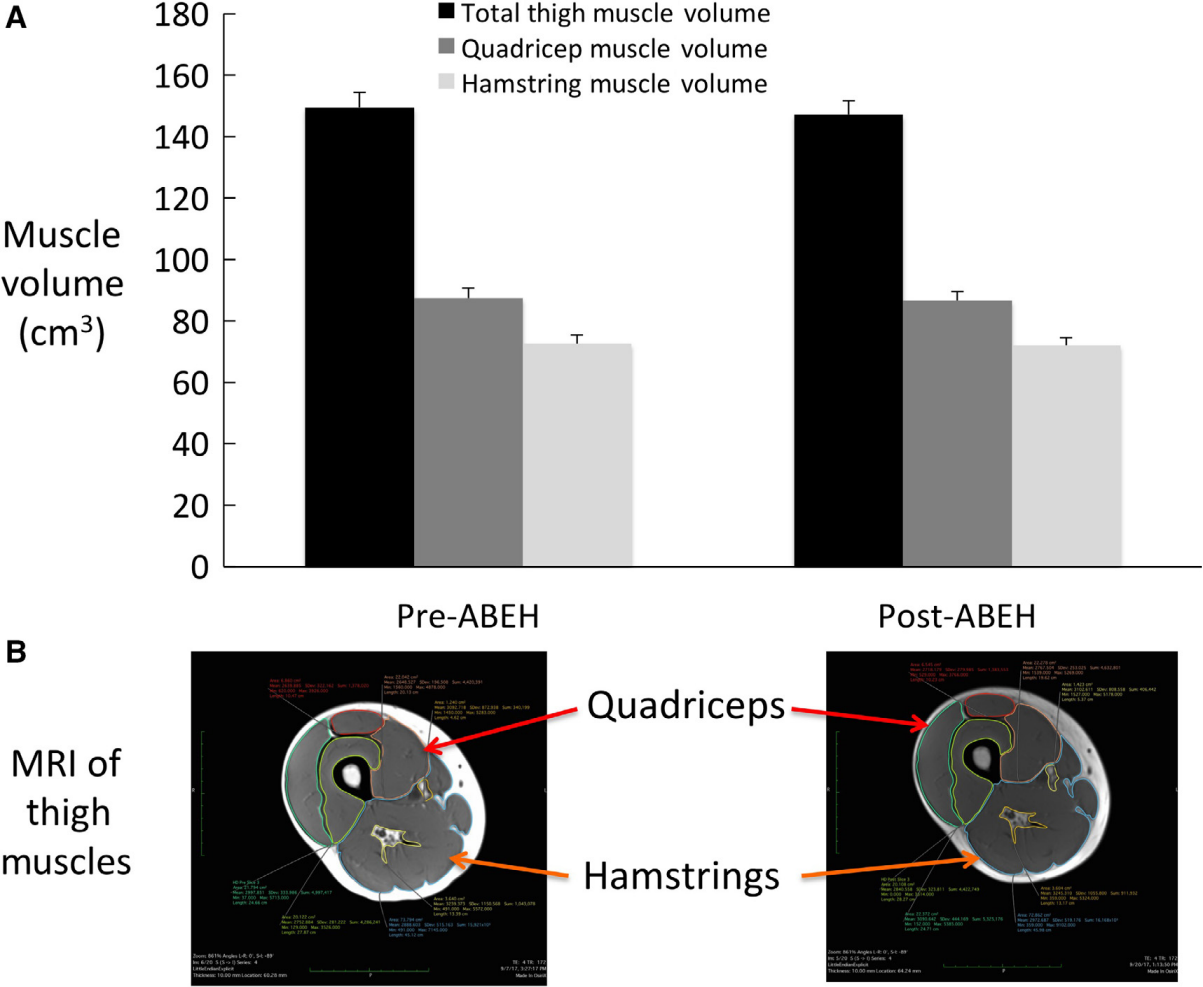
(A) Pre‐ and post‐ABEH measurements of total thigh muscle, quadricep muscle, and hamstring muscle volume via magnetic resonance imaging. Data are presented as mean±SD. (B) Representative molecular resonance images of pre‐ABEH and post‐ABEH in participant #1.

Total body weight and body mass index were reduced from pre‐ and post‐ABEH (Table 2). Overall, there were no changes in whole body, trunk LTM, arm LTM, or leg LTM (Tables 3 and 4). There were no differences in XT from pre‐ to post‐ABEH (Fig. 2A and B). Total FM, arm FM, leg FM, trunk FM, and visceral fat volume were reduced from pre‐ to post‐ABEH (Table 4). There were absolute reductions in LTM and XT in participant #1, with the highest pre‐ABEH amounts of LTM and XT. These variables either remained the same or slightly increased from pre‐ to post‐ABEH in participants #2, #3, and #4. The most provocative reductions in FM occurred in participants #2, #3, and #4, as the pre‐ABEH total fat mass of participant #1 was 11 kg lower by comparison.

**Table 3:** Body composition (lean)

	Pre	Post	**∆**
Total lean tissue mass (kg)			
#1	72.1	71.2	−0.9
#2	58.7	59.6	0.8
#3	74.8	75.3	−0.5
#4	64.3	64.2	−0.1
Mean ± SD	67.5 ± 6.4	67.6 ± 6.1	0.1 ± 0.7
Trunk lean tissue mass (kg)			
#1	32.6	31.6	−1.0
#2	27.4	27.4	0.1
#3	35.0	34.6	0.1
#4	29.9	29.4	−0.4
Mean ± SD	31.1 ± 2.7	30.8 ± 2.7	−0.3 ± 0.4
Leg lean tissue mass (kg)			
#1	24.8	24.4	−0.4
#2	19.7	20.1	0.5
#3	24.9	25.5	0.6
#4	22.2	22.6	0.4
Mean ± SD	22.9 ± 2.2	23.2 ± 2.1	0.3 ± 0.4
Arm lean tissue mass (kg)			
#1	10.9	11.3	−0.4
#2	8.3	8.6	0.3
#3	11.4	11.3	−0.1
#4	8.7	8.8	0.7
Mean ± SD	9.8 ± 1.4	10.0 ± 1.3	0.2 ± 0.2

Data are presented as means ± SD.

**Table 4:** Body composition (fat)

	Pre	Post	∆
Total fat mass (kg)			
#1	13.6	10.9	−2.7
#2	23.7	20.4	−3.3
#3	27.2	23.3	−3.9
#4	25.3	21.9	−3.4
Mean ± SD	22.4 ± 5.2	19.3 ± 4.9	−3.3 ± 0.2^a^
Trunk fat mass (kg)			
#1	7.4	5.4	−1.9
#2	13.4	11.4	−2.0
#3	18.4	15.5	−2.9
#4	14.7	12.4	−2.3
Mean ± SD	13.5 ± 4.0	11.2 ± 3.7	−2.3 ± 0.4^a^
Arm fat mass (kg)			
#1	1.8	1.5	−0.3
#2	2.5	2.2	−0.3
#3	3.1	2.7	−0.5
#4	2.8	2.4	−0.4
Mean ± SD	2.6 ± 0.5	2.2 ± 0.4	−0.4 ± 0.1^a^
Leg fat mass (kg)			
#1	3.5	3.0	−0.5
#2	6.9	5.9	−1.0
#3	4.6	4.4	−0.2
#4	6.8	6.1	−0.7
Mean ± SD	5.4 ± 1.5	4.9 ± 1.3	−0.6 ± 0.3^a^
Visceral fat mass (kg)			
#1	0.6	0.6	0.0
#2	1.1	0.7	−0.4
#3	2.0	1.6	−0.4
#4	0.8	0.7	−0.1
Mean ± SD	1.1 ± 0.5	0.9 ± 0.4	−0.2 ± 0.2^b^
Visceral fat volume (cm^3^)			
#1	618	610	−8
#2	1182	790	−392
#3	2170	1684	−486
#4	866	709	−157
Mean ± SD	1209 ± 590	948 ± 430	261 ± 188^b^

Data are presented as means ± SD.

a Represents significant difference (*P* < 0.05).

b Represents a trend toward significance (*P* < 0.05−0.100).

### Blood analysis

There were no changes in blood cholesterol, HDL‐cholesterol, or VLDL‐cholesterol from pre‐ to post‐ABEH. Despite only two participants (#1 and #2) completing blood sampling and analysis due to logistical complications with bush travel, there was strong trend (*P* = 0.055) toward a reduction in LDL‐cholesterol (Table 5).

**Table 5:** Blood lipids (*n* = 2)

	Pre	Post	∆
Total cholesterol (mg/dL)			
#1	176	193	17
#2	182	154	−28
Mean ± SD	179 ± 3	174 ± 20	−6 ± 23
LDL cholesterol (mg/dL)			
#1	50	23	−27
#2	110	91	−19
Mean ± SD	80 ± 30	57 ± 34	−23 ± 4^a^
HDL cholesterol (mg/dL)			
#1	113	160	47
#2	38	49	11
Mean ± SD	76 ± 38	105 ± 56	29 ± 18
Triglyceride (mg/dL)			
#1	66	48	−18
#2	172	72	−100
Mean ± SD	119 ± 53	60 ± 12	−59 ± 41
VLDL cholesterol (mg/dL)			
#1	10	13	3
#2	34	14	−20
Mean ± SD	22 ± 12	14 ± 1	−9 ± 12

Data are presented as means ± SD.

a Represents *P* = 0.06.

### Intrahepatic lipid

**Figure 3:**
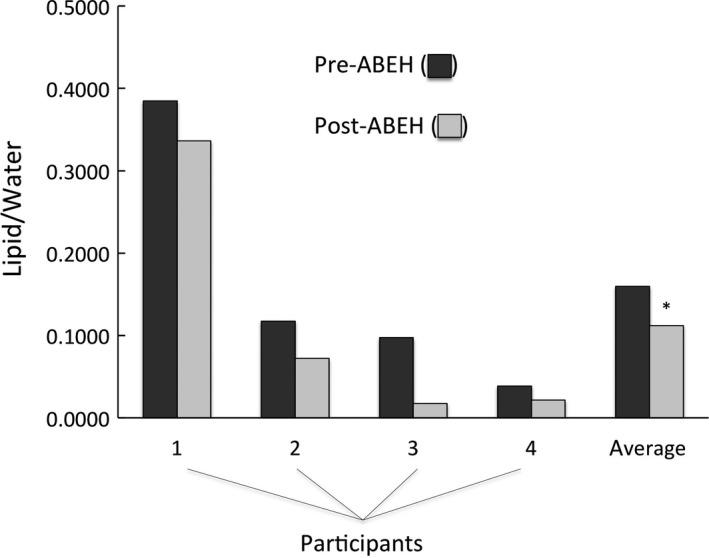
Individual and mean IHL as measured by magnetic resonance imaging/spectroscopy in four participants pre‐ and post‐ABEH. Units of IHL are expressed in relation to a standard of Intralipid. *Represents significant difference (*P* < 0.05).

There were significant reductions in IHL from 0.16 ± 0.13 to 0.11 ± 0.13 that represented consistent reductions in IHL across all four participants (Fig. 3).

## Discussion

Archaeological evidence supports the widespread existence of the hunter‐gatherer lifestyle throughout the Paleolithic period (Holt and Formicola 2008). Migrating across the land bridge (Beringia) that connected Asia to North America despite remarkable environmental and physical obstacles, these humans subsisted on populations of game and fish, surviving in what is now Alaska (Mulligan and Szathmáry 2017). While the Paleo diet has received a great deal of attention with regard to potential benefits on metabolic health, survival during the Paleolithic period was dependent upon large investments in physical activity. We hypothesized that the similarity of this practice in modern times would promote substantial rates of TEE linked to rapid and beneficial changes in body composition and IHL, aerobic capacity, and blood lipids in middle‐aged, relatively active male participants. Based on this experimental paradigm, it seems that an energy deficit may have fostered overall reductions in FM, IHL, and preservation of skeletal muscle.

When exercise training has been utilized to promote chronic negative energy balance in obese individuals, favorable alterations in body composition and metabolic risk factors have been demonstrated even without large amounts of weight loss (Donnelly et al. 2003, 2012; Coker et al. 2009; Jakicic et al. 2015; Ma et al. 2017). Exercise‐induced weight loss in middle‐aged individuals may even optimize reductions in visceral fat and improvements in hepatic insulin sensitivity exceeding that demonstrated by equivalent amounts of weight lost via caloric restriction (Coker et al. 2009). These beneficial adaptations are quite relevant as the accumulation of visceral fat and excess deposition of IHL have been shown to be closely related to the development of type 2 diabetes (Brouwers et al. 2016). Several studies have also shown that exercise training (resistance and aerobic) fosters 10–30% reductions in IHL with 4–16 week interventions (Brouwers et al. 2016). The consistent modifications in body composition were most likely linked to an increase in the mobilization of IHL and utilization of plasma free fatty acids due to negative energy balance derived from physical activity. By the most applicable comparison to our present study, metabolic stress is still evident in Hadza hunter‐gatherers, but their estimated level of energy expenditure was ~40% less than reported here (Pontzer et al. 2015).

While our study also demonstrated the favorable influence of physical activity and negative caloric balance on visceral fat and IHL, there are some important differences. These include the short‐term nature of our ABEH scenario, and the measurement of TEE and TEI that allowed us to estimate an average negative energy balance of ~9.0 MJ per day. We are not aware of any exercise training study that elicited this degree of negative energy balance and significant reductions in visceral fat and IHL over a very short‐term period. Given the resistance of visceral fat to the anti‐lipolytic influence of insulin (Ibrahim 2010), reductions in visceral fat may have lowered portal levels of free fatty acids. The increased utilization of free fatty acids would have then provided mutual benefits toward the reduction in IHL. These changes occurred in non‐obese individuals with modest amounts of visceral fat and IHL and a relatively low risk of cause‐specific mortality (Prospective Studies Collaboration, 2009). It is also important to mention very minimal alterations in FM in participant #1, who had the lowest FM and highest LTM prior to ABEH.

Despite a trend for a decrease in total body weight and BMI and significant reductions in adipose tissue and IHL, lean tissue mass and XT were preserved overall. This is consistent with studies that have demonstrated an increase in basal and aerobic exercise‐stimulated increments in protein synthesis (Short et al. 2004; Fujita et al. 2007). Acute bouts of resistance exercise have also been shown to promote elevations in protein synthesis that persist up to 48 h following cessation of the initial overload stimulus (Phillips et al. 1997). Regardless of the mode of exercise, mechanotransduction or mechanical stress on the contractile elements of the muscle promotes anabolic signaling and increased muscle protein synthesis (Hornberger et al. 2004). The data from these studies have been largely derived from tracing the incorporation of dietary amino acids into whole body and muscle protein synthesis using stable isotope methodology (Phillips et al. 1997). While invaluable in furthering our understanding of the adaptive responses to exercise, these types of studies are typically conducted under conditions of positive or stable caloric balance.

Our previous work has illustrated the retention of lean tissue mass under high levels of physical stress, even under conditions that would likely invoke a significant caloric imbalance (Coker et al. 2017; Johannsen et al. 2018). Our current data are consistent with and now potentially linked to the impressive degree of robusticity that was likely part of the caloric deficient Paleolithic period (Trinkhaus 2005). It is also interesting that LTM and XT decreased in participant #1 who had the highest amount of LTM and lowest FM. Participant #1 was the oldest (46 years of age) compared to other participants (40–41 years of age). It is unlikely that modest differences in age played a role in this difference but was linked to existing absolute differences in aerobic fitness and lean/fat ratio (Mikkelsen et al. 2013). On the other hand, chronic elevations in physical activity, when combined with energy deficit, may increase the need for dietary protein due to increased muscle proteolysis (Pasiakos et al. 2015). Future studies have been planned to explore the mechanisms responsible for potential variations in protein metabolism in this cohort and remains an area of interest beyond the laboratory setting (Pasiakos et al. 2015).

A recent meta‐analysis evaluated the influence of several short‐term (≤2 weeks) high intensity interval training (HIIT) periods and found mixed results with respect to improvements in aerobic capacity (Batacan et al. 2017). This particular analysis of the highly touted HIIT strategy focused primarily on the beneficial influence of high‐intensity interval training. Recognizing that the physical activity inherent with the ABEH would be quite difficult to duplicate in an urban setting, the length of the overload stimulus and modalities in the present study may have been somewhat similar to HIIT. For example, the harvest of three animals required multiple sequences of shared load carriage from harvest site to rafts and offloading of meat each evening to ensure adequate air circulation and dry storage. The harvests took place midway through the excursion on both hunts. The average and combined load carriage for all four participants would have been no less than 650 kg or 160 kg/person, while traversing 100 m to 500 m of difficult terrain at least twice a day. These activities were combined with significant periods of light, moderate, and vigorous activities that have been shown to positively influence oxidative metabolism (Keren and Epstein 1981; Kubukeli et al. 2002). While relative VO_2max_ displayed some improvement, alterations were not significant and were likely influenced by reductions in body weight. It is possible that 12 days of combined load carriage and chronic physical activity may positively influence oxidative metabolism, but we will need to evaluate this parameter using a larger sample size with a higher level of specificity. It is noteworthy that water turnover data during ABEH was considerably less than reported in studies with wildland firefighters even though the levels of TEE seem quite similar. This is likely due to differences in environmental conditions that necessitate higher levels of water intake in wildland firefighting compared to the ABEH model (Ruby et al. 2002).

The limitations of our study are largely linked to a small sample size of four participants. This was further complicated by the need to evaluate changes in background H_2_O enrichments and the lack of blood samples from two participants due to bush plane availability and weather conflicts. Without this correction, we would have missed alterations in background enrichment in participant #3, altering the average TEE by ~350 kcal (8%). With just two individuals completing the blood sampling, LDL‐cholesterol fell similarly in participants #1 and #2 with pre‐ABEH total cholesterol values below 200 mg/dl. While it is understood that exercise and weight loss has a favorable influence on LDL‐cholesterol (Batsis et al. 2017), we are only aware of one study that reported a decrease in LDL‐cholesterol over a short term (i.e., ≤2 week) period in healthy individuals (Bemelmans et al. 2010). The measurement of energy expenditure in all four participants was not possible due to our need to determine potential variations in background enrichments in participants from the lower 48 compared to participants from Alaska (Schoeller et al. 1986).

There are also some important limitations with regard to the calculation of TEE and TEI. For example, the estimation of TEI based on dietary recall may be underestimated by as much as 20% (Capling et al. 2017). Despite this potential variation, we were limited to the use of dietary recall. To reduce deficiencies in reporting, we included video and written documentation by our participants. Study participants consumed only the pre‐packaged foods that were transported into the backcountry with them. This approach parallels the concept of pre‐packaged meals in clinical research to document TEI with increased accuracy. The application of the DLW method is limited by an inability to calculate energy expenditure on a daily basis. On the other hand, the overall influence of alterations in activity, physical overload, and nutrient intake on body composition can be accurately assessed over a longer period (Margolis et al. 2014).

In conclusion, the obesity epidemic has now reached the entire world (Pedersen 2009; Prospective Studies Collaboration, 2009; Mitchell et al. 2011). The results of this initial study coincide with dramatic advances in technology that have included the agricultural and industrial revolutions, and the digital age (O'Keefe et al. 2011). These factors have modified and largely eliminated the demand for physical work or activity. Our results seem to suggest a potential link between modernization and the emerging diseasome that includes a cluster of diseases and conditions such as type 2 diabetes, cardiovascular disease, cancers, cognitive impairment, and depression (Pedersen 2009). The incidence of abdominal obesity and its connection to elevations in IHL and systemic inflammation represent the hallmarks of this modern diseasome. We used a combined approach, utilizing the advantage of a “real‐world” field setting and state‐of‐the‐art isotopic methodology and molecular imaging. We have demonstrated that activity patterns likely common during the Paleolithic period not only provided benefits with regard to nutritional intake, but may have also provided an activity overload stimulus that would have quickly reversed the etiology of metabolic disease (Mulligan and Szathmáry 2017). Future studies are needed in a larger cohort that also includes females and males. These data further emphasize that obesity in our modern society may simply be an adaptive response to excess nutrient availability relative to modest physical exertion.

## Conflict of Interest

Coker is a Managing Partner and Co‐Owner of Essential Blends, LLC that has received funding from the National Institutes of Health to develop clinical nutrition products. The data presented in this manuscript are unrelated.
